# A Time-Domain Perspective on the Structural and Electronic Response in Epitaxial Ferroelectric Thin Films on Silicon

**DOI:** 10.1021/acs.nanolett.4c00712

**Published:** 2024-07-23

**Authors:** Christelle Kwamen, Matthias Rössle, Wolfram Leitenberger, Pedro Rojo Romeo, Bertrand Vilquin, Catherine Dubourdieu, Matias Bargheer

**Affiliations:** † Helmholtz-Zentrum Berlin für Materialien und Energie, Wilhelm-Conrad-Röntgen Campus, BESSY II, Albert-Einstein-Str. 15, 12489 Berlin, Germany; ‡ Institut für Physik and Astronomie, Universität Potsdam, Karl-Liebknecht-Str. 24-25, 14476 Potsdam, Germany; § Université de Lyon, Institut des Nanotechnologies de Lyon (UMR5270/CNRS), Ecole Centrale de Lyon, 36 Avenue Guy de Collongue, 69134, Ecully Cedex, France; ∥ Helmholtz-Zentrum Berlin für Materialien und Energie, Institute Functional Oxides for Energy Efficient Information Technology (IFOX), Hahn-Meitner-Platz 1, 14109 Berlin, Germany; ⊥ Freie Universität Berlin, Physical and Theoretical Chemistry, Arnimallee 22, 14195 Berlin, Germany

**Keywords:** thin films, ferroelectrics, time-resolved X-ray diffraction, structural dynamics, hysteresis

## Abstract

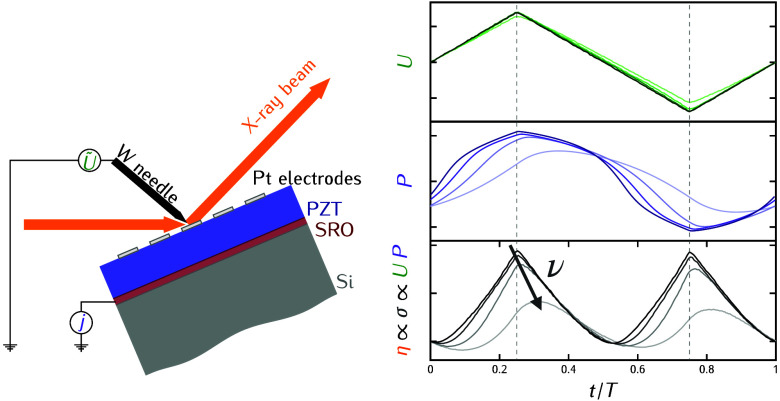

This operando study of epitaxial ferroelectric Pb(Zr_0.48_Ti_0.52_)O_3_ capacitors on silicon substrates studies their structural response via synchrotron-based time-resolved X-ray diffraction during hysteresis-loop measurements in the 2–200 kHz range. At high frequencies, the polarization hysteresis loop is rounded and the classical butterfly-like strain hysteresis acquires a flat dumbbell shape. We explain these observations from a time-domain perspective: The polarization and structural motion within the unit cell are coupled to the strain by the piezoelectric effect and limited by domain wall velocity. The solution of this coupled oscillator system is derived experimentally from the simultaneously measured electronic and structural data. The driving stress σ_FE_(*t*) is calculated as the product of the measured voltage *U*(*t*) and polarization *P*(*t*). Unlike the electrical variables, σ_FE_(*t*) and η(*t*) of the ferroelectric oscillate at twice the frequency of the applied electrical field. We model the measured frequency-dependent phase shift between η(*t*) and σ_FE_(*t*).

Ferroelectric (FE) thin films are technologically important and are found in different applications in our daily lives. For example, FEs are used for nonvolatile memories because of the ability to switch their polarization under an applied electric field or for sensor and actuator devices exploiting the strong coupling of electric field *E* and mechanical strain η.^1−3^ These physical properties scale with the device size and are strongly frequency-dependent.^4−6^ From an application standpoint, there is a growing interest in using ferroelectrics monolithically integrated on silicon since this is a CMOS-based technology platform. In the past 15 years, many efforts have been devoted to the epitaxial growth of various perovskite ferroelectrics such as BaTiO_3_, PbTiO_3_, or Pb(Zr_1–*x*_Ti_*x*_)O_3_ on silicon or GaAs.^7−12^ The ferroelectric lead-based solid solution Pb(Zr_1–*x*_Ti_*x*_)O_3_ is of particular interest due to its large piezoelectric coefficients.^13^ Enhanced piezoelectric properties are observed at the morphotropic phase boundary in the composition range 0.47 < *x* ≤ 0.52 that separates a Ti-rich tetragonal from a Zr-rich rhombohedral phase by a monoclinic intermediate phase.^14−18^ Few groups have reported the integration of epitaxial lead titanate films into capacitors on silicon substrates.^10,18−21^ Advantages of epitaxial films over polycrystalline ones include a well-defined polar axis and a smaller thickness (for achieving the same remanent polarization) thus requiring a lower voltage for polarization switching. For device applications, key features are the dynamics of the polarization switching and of the coupled electromechanical response. In FE devices, the dynamical response spans the wide time range from subpicosecond time scales^22,23^ over nanoseconds for domain dynamics.^24−29^ The switching dynamics in epitaxial films has been widely studied in micrometer-scale capacitors and is well described by the Kolmogorov–Avrami–Ishibashi (KAI) model.^30−33^ In this model, the switching kinetics is governed by the dynamics of domain nucleation, growth, and coalescence, assuming nucleation of domains at independent nucleation centers. For FE thin films, *in situ* synchrotron X-ray diffraction has become available to quantify the electromechanical response and fatigue.^34−36^ Few experiments are reported on single-crystalline films with the *c*-axis oriented perpendicular to the substrate surface.^14,24,37,38^ The simultaneous characterization of strain and polarization reported for ceramics^39^ was extended to thin films.^27,28,40^ However, to the best of our knowledge, time-resolved studies of the coupled dynamics of polarization and strain in ferroelectrics integrated on silicon and systematic experimental studies on the frequency-dependent structural response have not yet been reported.

In this paper, we report an *operando* study of the electro-mechanical coupling and its dynamics determined by simultaneous synchrotron X-ray diffraction and hysteresis-loop measurements at various frequencies of a thin epitaxial ferroelectric Pb(Zr_0.48_Ti_0.52_)O_3_ (PZT) film deposited on (001) Si and sandwiched between two metallic electrodes. The PZT film composition was chosen to be within the morphotropic phase boundary. We measure the time-dependence of the ferroelectric polarization *P*(*t*) and of the lattice strain η(*t*) of the PZT film for frequencies varying from 2 up to 200 kHz. We discuss the complex phenomenon of the periodically driven modulation of the ferroelectric polarization coupled to the structural deformation in terms of oscillator equations, which describe the dielectric displacement and the motion of the atoms within the unit cell along the FE soft mode.^41^ We find that the piezoelectric driving stress σ_FE_(*t*) ∝ *U*(*t*)*P*(*t*) oscillates at twice the frequency of the driving voltage *U*(*t*). We show that the phase delay ϕ_σ–η_ between the stress and the strain increases with the driving frequency, and we demonstrate that this phase lag contributes to the rounding of the structural η–*U* and electrical *P*–*U* hysteresis loops of the FE at high frequencies.

A 200-nm-thick epitaxial (001) oriented Pb(Zr_0.48_Ti_0.52_)O_3_ (PZT) film with a chemical composition reflecting that of the MPB of the Pb–Zr–Ti–O phase diagram was deposited by RF magnetron sputtering onto a sputtered 30-nm-thick epitaxial SrRuO_3_ (SRO) bottom electrode on a SrTiO_3_ epitaxial seed layer grown by molecular beam epitaxy on a (001) Si substrate. The details of all deposition processes can be found in refs (42−45). After the PZT deposition, in order to crystallize the FE film, the sample was flash-annealed at 650 °C for one min under an oxygen atmosphere. Circular Pt top electrodes with radii between 50 and 300 μm were then deposited by sputtering and structured using a UV photolithography lift-off process.^46^

The time-resolved ultrafast X-ray diffraction measurements under an applied electrical field were performed at the KMC-3 XPP^47^ endstation of the storage ring BESSY II, Berlin, Germany, operated in hybrid mode.^48^ The results presented in this paper were obtained on electrodes with a diameter of 300 μm, which corresponds to an area of ∼7 × 10^–4^ cm^2^. The size of the electrodes was chosen such that only one capacitor at a time was illuminated by the X-ray focus. We reproducibly reached life times of more than 10^7^ switching cycles on different electrodes. We applied a triangular voltage *U*(*t*) with different frequencies ν (2–200 kHz) and a peak voltage of *U*_max_ = ±7 V, which corresponds to an electric field strength of *E* = 350 kV/cm, well beyond the coercive field *U*_c_. The triangular voltage was generated by using a Keithley 3390 Arbitrary Function Generator. We contacted a single electrode with a tungsten needle with tip diameter of 5 μm and used silver paint to contact the bottom electrode in order to apply the field across the PZT layer as described in refs (27, 28, and 47). An Agilent DSO9404A oscilloscope with an input impedance of 50 Ω was used to record the switching current, *I*(*t*), and applied voltage, *U*(*t*), across the PZT film during the X-ray diffraction measurement. The polarization was obtained by numerically integrating the measured current, *I*(*t*), over time. A schematic electrical connection scheme is shown in Figure 1a. Monochromatic X-ray photons with an energy of 9 keV were detected by a fast scintillator with a decay time of ∼5 ns combined with a photomultiplier (Hamamatsu H7844). The photomultiplier was read out in single photon counting mode using a time-correlated single photon counter (PicoHarp300, PicoQuant) with an acquisition time window of up to 33 μs.^47^ Asymmetrically scattered X-rays were blocked by a vertical slit of approximately 1 mm width in front of the detector opening. We performed symmetric ω/2θ scans with ω = θ around the 002 out-of-plane Bragg reflection of PZT. The very good crystalline quality of the PZT and SRO films on Si was characterized by static X-ray diffraction with a Pilatus 100k area detector (Dectris) at the same photon energy. The reconstructed reciprocal space map in Figure 1b shows the 002 reflections of PZT and SRO, respectively. The ferroelectric film is oriented with its *c* axis out of plane, with negligible X-ray diffraction from potential 90° domains. The time-dependent strain η(*t*) was calculated from the shift of the 002 reflection of PZT along the PZT *c* axis as η(*t*) = (*c*(*t*) – *c*(*t* = 0))/*c*(*t* = 0). All experiments were performed at room temperature.

**Figure 1:**
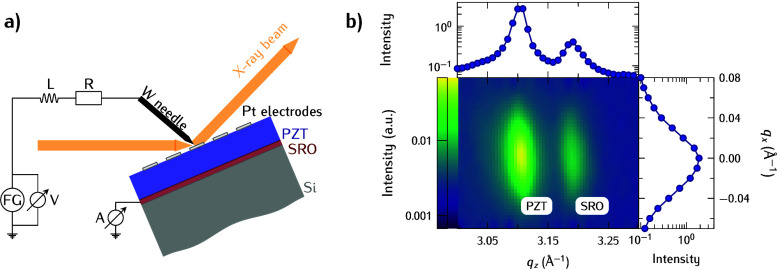
(a) Schematic electrical circuit of the connected sample (FE) during the measurement and that later is used for the modeling of the response. “FG” denotes the function generator that generates the driving sawtooth voltage, and “V” and “A” represent the two used input channels of the oscilloscope. A voltage amplifier is optional and was only used for low-frequency measurements. (b) Reciprocal space map of the 0 0 2 reflections of PZT and SRO respectively in the pristine state without an applied field. The solid symbols are obtained by the integration along the *q*_*x*_ and *q*_*z*_ directions, respectively.

We first present the frequency-dependent electrical and structural data as *P*–*U* and η–*U* hysteresis loops in Figure 2. In Figure 2a, we show the *P*–*U* hysteresis loops for frequencies ν ≤ 20 kHz, which are open saturated loops as expected for a ferroelectric material.^49^ The remnant polarization is rather low, which indicates considerable back switching of domains after saturation. A PUND analysis (not shown) reveals a very small dielectric charging and discharging current with an RC time constant of about 0.1 μs. In addition, large switching currents flow for about 25 μs even for each second Up (or Down) pulse of the PUND sequence. Such slowly responding currents cannot fully change the polarization in a short time, i.e., at high frequencies. As ν is increased from 10 up to ∼40 kHz (Figure 2a and b), the coercive field *U*_c_ increases by almost a factor of 2, consistent with literature results.^50,51^ The double logarithmic plot of *U*_c_ versus ν in Figure 2e represents a power law *U*_c_ ∝ ν^β^ with β = 0.33 = *D*/6 over the whole range of ν, which is consistent with a dimensionality *D* = 2 of the domain growth in thin epitaxial films as given by the KAI model.^33,50−54^ The *P*–*U* loops at frequencies ν ≥ 20 kHz show in contrast rounded shapes, and the absence of saturation, which might originate from leakage due to mobile defects like oxygen vacancies in FEs.^51,53,55^ However, such leakage should occur preferentially at low frequencies, where the leakage current flows in one direction for a long time and may decrease at high frequency, where also the remnant polarization is smaller because some domains are not switched fast enough.^56^ At high frequencies, the timespan where the applied voltage exceeds the coercive field is increasingly short. This effect is enhanced, because the coercive voltage increases. Figure 2c and d show that the butterfly loops η–*U* are also simultaneously rounded as the *P*(*E*) loops and finally adopt a dumbbell shape where the maximum strain occurs when the driving voltage is already ramped down. This implies that the FE layer is still expanding while the applied voltage is already reduced and that the FE reaches its maximum expansive strain considerably *after* the driving voltage *U*(*t*) has reached its maximum value. Simultaneously, the saturation polarization *P*_sat_ is reduced. In Figure 2f, clearly two regimes can be distinguished: For ν ≤ 20 kHz, the frequency dependence of *P*_sat_ follows a −ν dependence, whereas at ν ≥ 20 kHz, the polarization is inversely proportional to the frequency ν^–1^. The frequency at which the crossover occurs coincides with the frequency at which the pronounced rounding of the hysteresis loops in Figure 2a–d is observed.

**Figure 2:**
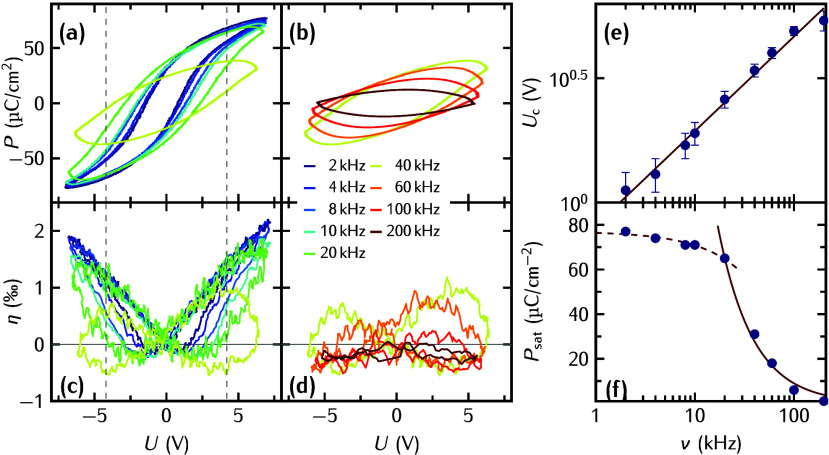
(a–d) Simultaneously measured *P*–*U* and η–*U* loops at different frequencies ν of the applied sawtooth pulse sequence. Panels a and b show the *P*–*U* loops for ν < 40 kHz and ν ≥ 40 kHz, respectively. In panels c and d, we show the corresponding η–*U* loops. In e, we plot *U*_C_ (filled circles) from the *P*–*U* loops shown in a and b on a *double* logarithmic scale as a function of ν, and the solid line is a fit to the data using ν^β^ with β = 0.33. (f) Plot of the saturation polarization *P*_sat_ (filled circles) on a *semi*-logarithmic scale as a function of ν, and the dashed line indicates a fit to the data assuming −ν, whereas the solid line indicates a fit using ν^–1^.

In the time-domain perspective displayed in Figure 3, the measured polarization *P*(*t*) (panel b), which is derived from the current, oscillates in phase with *U*(*t*) (panel a) at low frequencies. At frequencies ν ≥ 40 kHz, *P*(*t*) is phase-shifted with a value of ϕ_*P*–*U*_ = −π/2. This explains the rounded hysteresis loop because the highest polarization occurs later than the highest voltage. In Figure 3d, we quantify the strain η via time-resolved X-ray diffraction, which measures the absolute values of the *c* axis lattice parameter (right vertical axis). We note that at high frequencies the lattice constant reaches values that are smaller than those at any time for low frequency actuation. The lattice is compressed because the polarization and applied voltage are out of phase and hence there are times (e.g., *t*/*T* = 0.25 to 0.5) where polarization and voltage have opposite sign. This rationalizes the overall negative strain values at high frequency (Figures 2d).

**Figure 3:**
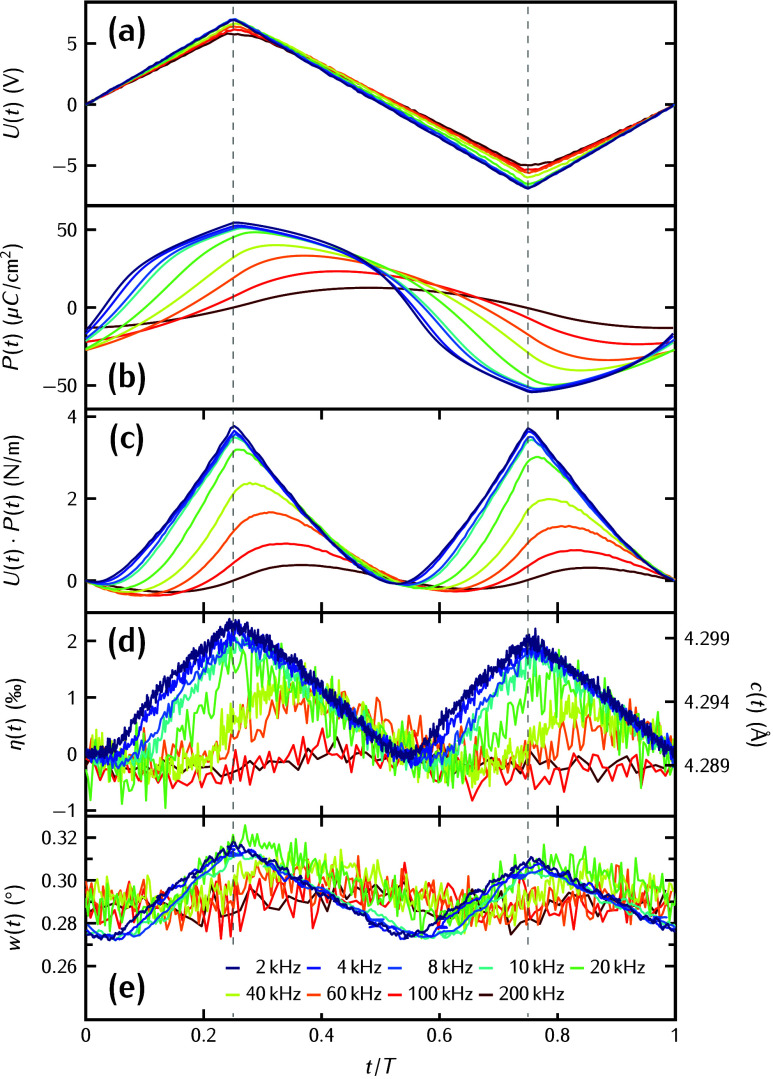
(a) Applied sawtooth voltage *U*(*t*/*T*) normalized by the period *T* of the frequency. (b) Measured polarization *P*(*t*/*T*) of the FE as obtained by the integraton of the switching current over time. (c) Product *U*(*t*/*T*)·*P*(*t*/*T*) that closely resembles the time-response of the measured transient strain η(*t*/*T*) of the FE shown in panel d. (e) Peak width *w*(*t*/*T*) of the PZT Bragg reflection.

Next, we show that the time-dependent piezoelectric stress σ_FE_ ∝ *P*(*t*) *U*(*t*) in Figure 3c is proportional to transient polarization *P*(*t*) and applied voltage *U*(*t*). The macroscopic polarization *P*(*t*) is proportional to the difference in the volume fractions *V*_up_ – *V*_down_ of the positively and negatively poled domains, which exhibit opposite piezoelectric effect. Hence, the piezoelectric coefficient can be approximated by using a time-dependent effective piezoelectric stress coefficient,^57^ which we model as *e*_33_(*t*) = *e*_33_^0^(2*V*_up_(*t*)/*V* – 1) = *e*_33_^0^(2*P*_up_(*t*)/*P*_sat_ – 1), as a first order approximation neglecting any domain wall contributions. This term is proportional to the volume fractions with up and down polarization, which are, in turn, described by the averaged polarization. If the entire film of thickness *d* is poled up, we find *e*_33_ = *e*_33_^0^ in this model, and if the entire film is poled down, it is *e*_33_(*t*) = −*e*_33_^0^. Thus, the piezoelectric stress
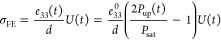
1acting on the ferroelectric crystal oscillates at twice the frequency of the driving voltage *U*(*t*) because the effective piezoelectric coefficient *e*_33_(*t*) changes its sign together with the voltage, albeit with a phase delay ϕ_*P*–*U*_ between *U*(*t*) and *P*(*t*). We observe that ϕ_*P*–*U*_ ≤ π/2 for the frequencies investigated in this work. The transient strain η(*t*) (see Figure 3d) essentially follows the driving stress with the doubled frequency and exhibits an additional phase shift ϕ_σ–η_ that increases with frequency.

In Figure 3e, we show how the peak width *w*(*t*) of the PZT 002 Bragg reflection changes for the different applied frequencies. The peak width at high frequencies has a large average value with only small modulation, indicative of a domain pattern with many small domains of up and down polarizations that slightly switch back and forth. At lower frequencies, the peak width strongly depends on the currently applied voltage since inhomogeneities of the capacitor dominate the variations of the local expansion.^24^

In the following, we relate the rounding of the hysteresis to the delayed maxima of the *P*–*U* loops described by the phase shift ϕ_*P*–*U*_ and the characteristic modification of the η–*U* loops to the concomitant stress σ_FE_(*t*) to which the strain η responds with an additional phase shift ϕ_σ–η_. To resolve this complex phenomenon of a periodically driven modulation of the FE polarization coupled to the structural deformation we adopt the model describing FE polarization using an oscillator model.^41^ We set up a system of differential equations for *P* and η: The voltages at each component in the circuit (see Figure 1a) add up to the external voltage supplied by the function generator: *U* = *U*_L_ + *U*_FE_ + *U*_R_. Using the inductance *L* and resistance *R* of the circuit including the wiring, the thickness *d* and dielectric function ε_FE_ of the FE capacitor, we can recast this equation in terms of the dielectric displacement *D* = ε_0_*E* + *P* starting from a classical damped harmonic oscillator:
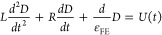
2

The strain η within the FE is driven by the same *U*(*t*); however, the stress σ_FE_ = *e*_33_(*t*) *U*(*t*)/*d* ∝ *P*(*t*) *U*(*t*)^57^ couples the differential equation for the strain to eq 2:
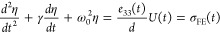
3where γ is an empirical damping constant and ω_0_ = 2*πν*_0_ is the mechanical angular eigenfrequency of the system. This system of equations challenges theoretical modeling because, in the time-dependent perspective, the FE properties are not constant anymore.^41^ For a full solution, the hysteretic behavior of ε_FE_(*t*) must be included, which necessarily adds a memory of the history of the sample. In the following, we use the measured polarization *P*(*t*) to rationalize the solution of eq 3. The time-dependent strain η(*t*) represented in eq 3 as a damped harmonic oscillator essentially follows the driving stress σ_FE_(*t*) at the second harmonic of driving voltage *U*(*t*). The increasing phase delay that η acquired with respect to σ_FE_ allows us to extract a mechanical damping constant that is connected to the viscous properties of the FE domain walls. The lowest mechanical resonance frequency ν_0_ = 1/*T* ∼ 10 MHz of the capacitor can be roughly estimated by the time *T* = 2*r*/*v*_s_ ≈ 0.1 μs it takes sound at velocity *v*_s_ ≈ 3.5 nm/ps^58^ to propagate through the diameter 2*r* = 300 μm of the electrode. The driving frequency ν = 1/*T*_*U*_ ≤ 200 kHz is much lower, as the sawtooth period *T*_*U*_ ranges between 5 and 500 μs. Therefore, we take the textbook result^59^ for the phase shift ϕ between η(*t*) and σ_FE_(*t*) of the solution to eq 3 and approximate it by
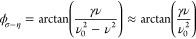
4

Figure 4 shows the excellent agreement of the phase lag determined from the experimental data shown in Figure 3 and the result of eq 4 with the damping γ = 1.5 GHz as the only fitting parameter. The result is shown by the filled circles in Figure 4 together with the fit to these points using eq 4 shown as a solid line.

**Figure 4:**
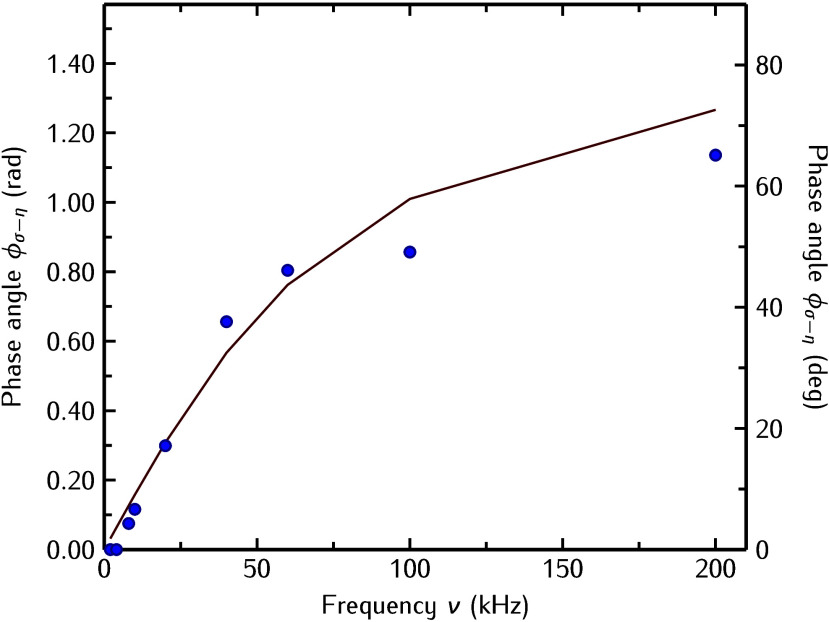
Phase angle ϕ_σ–η_ between the stress σ_FE_(*t*) and the strain η(*t*) as a function of frequency ν. The filled symbols are extracted from the experimental data, and the solid line is the result from the fit using eq 4 with γ = 1.5 GHz to the experimental data.

In conclusion, this time-domain perspective directly explains how the ferroelectric hysteresis loops in our samples are affected by different driving frequencies: (i) The rounding of the *P* −*U* hysteresis is caused by the phase lag between *P*(*t*) and *U*(*t*), which we relate mainly to the slow domain wall velocity.^24,28^ (ii) For lower frequencies, the polarization can follow the driving voltage, which results in the observed large average stress. (iii) The reduced maximum polarization at high frequencies originates from the fact that, at higher frequencies, not yet all domains are oriented along the applied field direction when the maximum voltage is reached. (iv) The negative average strain of the hysteresis at ν = 200 kHz is a consequence of the phase shifts that let *U*(*t*) and *P*(*t*), and hence the time-dependent piezoelectric stress coefficient *e*_33_(*t*), have opposite sign. (v) In part, the increasing coercive field *U*_c_ with increasing frequency is a result of the phase lag of the polarization, which implies that the zero crossing of the polarization is only reached later at higher voltage.

We believe that this study is an important contribution to the interpretation of hysteresis loops in the regime of high driving frequencies and hope to stimulate further theoretical and experimental work that accounts for the complex interplay of polarization and strain via domain-wall motion and viscoelasticity. In the context of application, our study pioneers strain analysis of ferroelectrics integrated on Si with time-resolved X-ray diffraction.

## Data Availability

Raw data were generated at the synchrotron storage ring BESSY II operated by the Helmholtz-Zentrum Berlin für Materialien and Energie, Germany, large scale facility. Derived data supporting the findings of this study are available from the corresponding authors upon reasonable request.
